# Recent Advances in the Chemical Composition of Propolis

**DOI:** 10.3390/molecules191219610

**Published:** 2014-11-26

**Authors:** Shuai Huang, Cui-Ping Zhang, Kai Wang, George Q. Li, Fu-Liang Hu

**Affiliations:** 1 College of Animal Sciences, Zhejiang University, Hangzhou 310058, China; E-Mails: asmallcaths@163.com (S.H.); lgzcplyx@aliyun.com (C.-P.Z.); kaiwang628@gmail.com (K.W.); 2 Faculty of Pharmacy, University of Sydney, Sydney, NSW 2006, Australia

**Keywords:** propolis, honeybee, flavonoids, phenypropanoids, terpenenes, plant origin

## Abstract

Propolis is a honeybee product with broad clinical applications. Current literature describes that propolis is collected from plant resins. From a systematic database search, 241 compounds were identified in propolis for the first time between 2000 and 2012; and they belong to such diverse chemical classes as flavonoids, phenylpropanoids, terpenenes, stilbenes, lignans, coumarins, and their prenylated derivatives, showing a pattern consistent with around 300 previously reported compounds. The chemical characteristics of propolis are linked to the diversity of geographical location, plant sources and bee species.

## 1. Introduction

Propolis is a honeybee product with a broad spectrum of biological properties [1]. As a resinous substance, propolis is prepared by the honeybees to seal the cracks, smooth walls, and to keep moisture and temperature stable in the hive all year around. Raw propolis is typically composed of 50% plant resins, 30% waxes, 10% essential and aromatic oils, 5% pollens and 5% other organic substances. It has been reported that propolis is collected from resins of poplars, conifers, birch, pine, alder, willow, palm, *Baccharis dracunculifolia*, and *Dalbergia ecastaphyllum* [2,3,4].

Propolis is widely used to prevent and treat colds, wounds and ulcers, rheumatism, sprains, heart disease, diabetes [5,6,7,8] and dental caries [9] due to its diverse biological properties such as anti-inflammatory [8,10,11,12], antimicrobial, antioxidant, antitumor [3], antiulcer and anti-HIV activities [13]. The wide application of propolis in modern medicine has drawn growing attention to its chemical composition. Many studies have revealed that the observed effects might be the result of synergistic action of its complex constituents [14,15,16]. 

**Figure 1:**
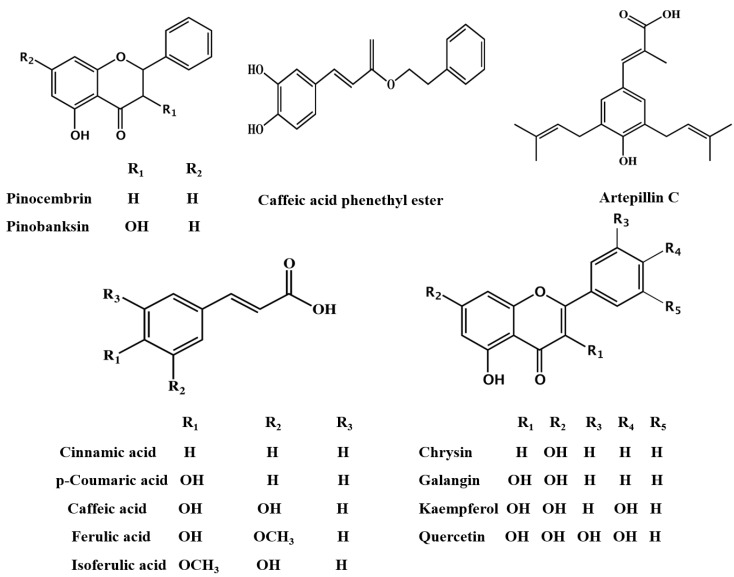
Representative chemical components in propolis.

Previous reviews [3,17,18] have covered the knowledge about the chemical composition and botanical origin of propolis throughout 20th century. Until 2000, over 300 chemical components belonging to the flavonoids, terpenes, and phenolics have been identified in propolis. Some representative chemical compounds are summarized in Figure 1. 

The characteristic constituents in temperate region propolis are flavonoids without B-ring substituents, such as chrysin, galangin, pinocembrin, pinobanksin. Caffeic acid phenethyl ester (CAPE) is a major constituent of temperate propolis with broad biological activities, including inhibition of nuclear factor κ-B; inhibition of cell proliferation; induction of cell cycle arrest and apoptosis. In tropical region propolis, especially Brazilian green propolis, the dominating chemical components are prenylated phenylpropanoids (e.g., artepillin C) and diterpenes. For propolis produced in the Pacific region, geranyl flavanones are the characteristic compounds which are also found in propolis from the African region [19].

The chemical composition of propolis is susceptible to the geographical location, botanical origin [20,21,22,23], and bee species [23]. In order to provide a theoretical basis for studying the chemical composition and pharmacological activity of propolis and plant sources, and controlling the quality, chemical components that were isolated for the first time from propolis between 2000 and 2012 were scouted and summarized from databases including BioMed Central, Biosis Citation Index, Medline, and PubMed. 

## 2. Chemical Compounds in Propolis

With the development of separation and purification techniques such as high performance liquid chromatography (HPLC), thin layer chromatography [24], gas chromatography (GC), as well as identification techniques, such as mass spectroscopy (MS) [25], nuclear magnetic resonance (NMR), gas chromatography and mass spectroscopy (GC-MS) [26], more compounds have been identified in propolis for the first time; including flavonoids, terpenes, phenolics and their esters, sugars, hydrocarbons and mineral elements. In contrast, relatively common phytochemicals such as alkaloids, and iridoids have not been reported. Two hundred and forty one (241) compounds have been reported for the first time from propolis between 2000 and 2012. Their chemical category, geographical locations, and possible plant source, are summarized below.

## 3. Flavonoids

As the major constituents of propolis, flavonoids contribute greatly to the pharmacological activities of propolis. The quantity of flavonoids is used as a criterion to evaluate the quality of temperate propolis [27]. Flavonoids have a broad spectrum of biological properties, such as antibacterial, antiviral and anti-inflammatory effects [16,28]. According to the chemical structure, flavonoids in propolis are classified into flavones, flavonols, flavanones, flavanonols, chalcones, dihydrochalcones, isoflavones, isodihydroflavones, flavans, isoflavans and neoflavonoids. From 2000 to 2012, 112 flavonoids were identified in different type of propolis for the first time (Table 1). In addition, flavonoid glycosides that are very rare in propolis were identified; they are isorhamnetin-3-*O*-rutinoside [29] and flavone *C*-glycoside [30].

Five flavones **1**–**5** were identified in Chinese, Polish, Egyptian and Mexican propolis. According to the geographical origin and the typical chemical compounds, the botanical origins of these propolis samples are assumed to be the genus *Populus.* In samples from the Solomon Islands and Kenya, researchers identified four flavonols **6**–**9** and confirmed that these compounds exhibited potent antibacterial activity [31]. The majority of the identified compounds were also found in the plants *Macaranga*, suggesting that the genus *Macaranga* is the likely plant source. In Pacific propolis, scientists identified many prenylated flavanones **21**–**31** which exhibited strong antimicrobial activity because the lipophilic prenyl group could rapidly damage the membrane and cell wall function [32]. Some flavanones **11**, **13**, **14**, **17**–**19** were also identified in poplar propolis. Sherstha *et al.* identified three flavanonols **42**–**44** in Nepalese propolis, Portuguese propolis and Australian propolis, respectively. 

**Table 1:** Flavonoids identified in propolis since 2000.

No.	Chemical Name	Geographical Location	Reference
Flavones			
1	Luteolin	China	[33]
2	6-Cinnamylchrysin	China	[34]
3	3',5-Dihydroxy-4',7-dimenthoxy flavone	Poland	[26]
4	Hexamethoxy flavone	Egypt	[35]
5	(7''*R*)-8-[1-(4'-Hydroxy-3'-methoxyphenyl) prop-2-en-1-yl]chrysin	Mexico	[36]
Flavonols			
6	2'-(8"-Hydroxy-3",8"-dimethyl-oct-2"-enyl)-quercetin	Solomon Island	[31]
7	8-(8"-Hydroxy-3",8"-dimethyl-oct-2"-enyl)-quercetin	Solomon Island	[31]
8	2'-Geranylquercetin	Solomon Island	[31]
9	Macarangin	Kenya	[37]
10	(7"*R*)-8-[1-(4'-Hydroxy-3'-methoxyphenyl)prop-2-en-1-yl]-galangin	Mexico	[36]
Flavanones			
11	3-*O*-[(*S*)-2-Methylbutyroyl]pinobanksin	China	[34]
12	(2*S*)-5,7-Dihydroxy-4'-methoxy-8-prenylflavanone	Solomon Island	[31]
13	Hesperitin-5,7-dimethyl ether	Portugal	[38]
14	Pinobanksin-5-methyl-ether-3-*O*-pentanoate	Portugal	[38]
15	7-*O*-Prenylstrobopinin	Greek	[39]
16	7-*O*-Prenylpinocembrin	Greek	[39]
17	(2*R*,3*R*)-3,5-Dihydroxy-7-methoxyflavanone 3-(2-methyl)-butyrate	Mexico	[36]
18	(2*R*,3*R*)-6[1-(4'-Hydroxy-3'-methoxyphenyl) prop-2en-1-yl] pinobanksin	Mexico	[40]
19	(2*R*,3*R*)-6[1-(4'-Hydroxy-3'-methoxyphenyl) prop-2en-1-yl]-pinobanksin-3-acetate	Mexico	[40]
20	3',4',6-Trihydroxy-7-methoxy flavanone	Nepal	[41]
21	5,7,3',4'-Tetrahydroxy-5'-*C*-geranylflavanone	Japan	[42]
22	5,7,3',4'-Tetrahydroxy-6-*C*-geranylflavanone	Japan	[42]
23	5,7,3',4'-Tetrahydroxy-2'-*C*-geranylflavanone	Japan	[42]
24	5,7,3',4'-Tetrahydroxy-2'-C-geranyl-6-prenlyflavanone	Japan	[42]
25	Propolin A	Taiwan	[43]
26	Propolin B	Taiwan	[43]
27	Propolin E	Taiwan	[43]
28	Sigmoidin B	Taiwan	[43]
29	Bonannione A	Taiwan	[31]
30	Solophenol A	Solomon Island	[31]
31	Sophoraflavanone A	Solomon Island	[31]
32	(2*S*)-7-Hydroxyflavanone	Brazil	[44]
33	(2*S*)-Liquiritigenin	Brazil	[44]
34	(2*S*)-7-Hydroxy-6-methoxyflavanone	Brazil	[44]
35	(2*S*)-Naringenin	Brazil	[44]
36	(2*S*)-Dihydrobaicalein	Brazil	[44]
37	(2*S*)-Dihydrooroxylin A	Brazil	[44]
38	(2*R*,3*R*)-3,7-Dihydroxyflavanone	Brazil	[44]
39	Garbanzol	Brazil	[44]
40	(2*R*,3*R*)-3,7-Dihydroxy-6-methoxyflavanone	Brazil	[44]
41	Alnustinol	Brazil	[44]
42	(2*R*, 3*R*)-3,6,7-Trihydroxyflavanone	Nepal	[41]
43	5-Methoxy-3-hidroxyflavanone	Portugal	[38]
44	5,7-Dihydroxy-6-methoxy-2,3-Dihydroflavonol-3-acetate	Australia	[45]
Isoflavones			
45	Odoratin	Nepal	[41]
46	7,3',4'-Trihydroxy-5'-methoxyisoflavonoid	Nepal	[41]
47	6,7,3'-Trihydroxy-4'-methoxyisoflavonoid	Nepal	[41]
48	7,3'-Dihydroxy-6,5'- methoxyisoflavonoid	Nepal	[41]
49	7-Hydroxy-4'-methoxyisoflavonoid	Cuba	[46]
50	5,7-Dihydroxy-4'-methoxyisoflavonoid	Cuba	[46]
51	Calycosin	Brazil	[44]
52	7,4'-Dihydroxyisoflavone	Brazil	[24]
53	Homopterocarpin	Brazil	[24]
54	Medicarpin	Brazil	[24]
55	4',7-Dimethoxy-2'-isoflavonol	Brazil	[24]
Isodihydroflavones			
56	Daidzein	Brazil	[44]
57	Formononetin	Brazil	[44]
58	Xenognosin B	Brazil	[44]
59	Biochanin A	Brazil	[44]
60	Pratensein	Brazil	[44]
61	2'-Hydroxybiochanin A	Brazil	[44]
62	(3*S*)-Vestitone-	Brazil	[44]
63	(3*S*)-Violanone	Brazil	[44]
64	(3*S*)-Ferreirin	Brazil	[44]
65	(3*R*)-4'-Methoxy-2',3,7-trihydroxyisoflavanone	Brazil	[44]
66	Biochanin	Cuba	[25]
Chalcones			
67	3,4,2',3'-Tetrahydroxychalcone	Brazil	[30]
68	Isoliquiritigenin	Brazil	[44]
69	4,4'-Dihydroxy-2'-methoxychalcone	Brazil	[44]
Dihydrochalcones			
70	(α*S*)-α,2',4,4'-Tetrahydroxydihydrochalcone	Brazil	[44]
71	2',4'-Dihydroxychalcone	Brazil	[44]
72	2',6'-Dihydroxy-4',4-dimethoxydihydrochalcone	Canada	[47]
73	2',4',6'-Trihydroxy-4-methoxydihydrochalcone	Canada	[47]
74	2',6',4-Tryhydroxy-4'-methoxydihydrochalcone	Canada	[47]
Flavans			
75	8-[(*E*)-4-Phenylprop-2-en-1-one]-(2*R*,3*S*)-2-(3,5-dihydroxyphenyl)-3,4-dihydro-2H-2-be-nzopyran-5-methoxyl-3,7-diol,	China	[48]
76	8-[(*E*)-4-Phenylprop-2-en-1-one]-(2*S*,3*R*)-2-(3,5-dihydroxyphenyl)-3,4-dihydro-2H-2-benzopyran-5-methoxyl-3,7-diol	China	[48]
77	8-[(*E*)-4-Phenylprop-2-en-1-one]-(2*R*,3*S*)-2-(3-methoxyl-4-hydroxyphenyl)-3,4-dihydro-2H-2-benzopyran-5-methoxyl-3,7-diol	China	[48]
78	3-Hydroxy-5,6-dimethoxyflavan	Mexico	[49]
Isoflavans			
79	(3*S*)-Vestitol	Brazil	[44]
80	(3*S*)-Isovestitol	Brazil	[44]
81	(3*S*)-7-*O*-Methylvestitol	Brazil	[44]
82	(3*S*)-Mucronulatol	Brazil	[44]
83	7,4'-Dihydroxy-2'-methoxyisoflavone	Cuba	[46]
84	Neovestitol	Cuba	[25]
Pterocarpins (a type of neoflavonoid)			
85	Medicarpin	Cuba	[46]
86	4-Hydroxymedicarpin	-	[46]
87	Homopterocarpin	Cuba	[46]
88	4'-Methoxy-5'hydroxyvesticarpan	-	[46]
89	3,8-Dihydroxy-9-methoxypterocarpan	Cuba	[46]
90	3-Hydroxy-8,9-dimethoxypterocarpan	Cuba	[46]
91	3,4-Dihydroxy-9-methoxypterocarpan	Cuba	[46]
92	3,10-Dihydroxy-9-methoxypterocarpan	Brazil	[44]
93	6a-Ethoxymedicarpin	Brazil	[44]
94	(6a*R*,11a*R*)-4-Methoxymedicarpin	Brazil	[44]
Open-chain neoflavonoids			
95	Neoflavonoid 1	Nepal	[50]
96	Neoflavonoid 2	Nepal	[50]
97	Neoflavonoid 3	Nepal	[50]
98	Neoflavonoid 4	Nepal	[50]
99	Neoflavonoid 5	Nepal	[50]
100	Neoflavonoid 6	Nepal	[50]
101	Neoflavonoid 7	Nepal	[50]
102	Neoflavonoid 8	Nepal	[50]
103	Neoflavonoid 9	Nepal	[50]
104	Neoflavonoid 10	Nepal	[50]
105	(*S*)-3'-hydroxy-4-methoxydalbergione	Nepal	[51]
106	(*S*)-3',4'-dihydroxy-4-methoxydalbergione	Nepal	[51]
107	(S)-4-methoxydalbergione	Nepal	[51]
Other flavonoids			
108	2,6-Dihydroxy-2-[(4-hydroxyphenyl)methyl]-3-benzofuranone	Brazil	[44]
109	2-(2',4'-Dihydroxyphenyl)-3-methyl-6-methoxybenzofuran	Brazil	[44]
110	1-(3',4'-Dihydroxy-2'-methoxyphenyl)-3-(phenyl)propane	Mexico	[49]
111	(*Z*)-1-(2'-Methoxy-4',5'dihydroxyphenyl)-2-(3-phenyl)propene	Mexico	[49]

**Figure 2:**
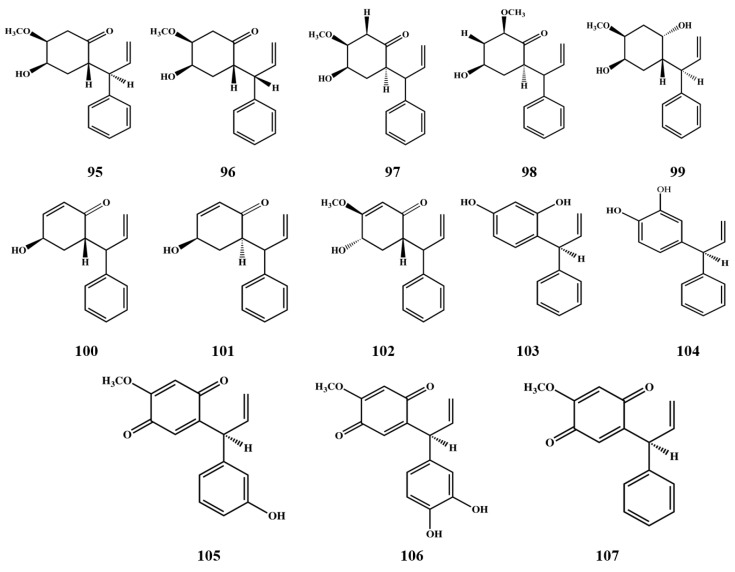
Open-chain neoflavonoids in propolis.

Red Brazilian propolis is a new type of propolis that has attracted wide attention. Researchers identified many compounds typically found in resinous exudates of leguminous plant (*Dalbergia ecastophyllum*) including 10 flavanones **32**–**41**, four isoflavones **51**–**55**, 11 isodihydroflavones **56**–**65**, three chalcones **67**–**69**, two dihydrochalcones **70**–**71**. Three dihydrochalcones **72**–**74** that are considered to be characteristic for the bud exudates of Tacamahaca poplars were found in Canadian samples for the first time. Sha *et al.* and Lotti *et al.* identified some flavans **75**–**78** with high cytotoxic activity in Chinese and Mexican propolis [48,49]. Piccinelli *et al.* identified two isoflavones: 7-hydroxy-4'-methoxyisoflavonoid and 5,7-dihydroxy- 4'-methoxy isoflavonoids in red Cuban propolis, although their plant source has not been confirmed. They presumably originated from Leguminous plants, which is the same botanical origin of red Brazilian propolis [46]. At the same time, isoflavanes **79**–**84** and pterocarpins **85**–**94** were also found in the two types of red propolis. In samples from Nepal, 14 unique open-chain neoflavonoids **95**–**107** (Figure 2) were identified, which are used as markers of the plant source of this type of propolis. 

Among the compounds isolated from Nepalese propolis, (*S*)-4-methoxydalbergione and obtusaquinol were reported as constituents of *Dalbergia* and *Machaerium* woods, but some neoflavonoids such as cearoin and 9-hydroxy-6,7-dimethoxydalbergiquinol were identified only in *Dalbergia* species [50]. Other flavonoids **108**–**111** found in Brazilian and Mexican propolis, respectively, are listed in Table 1.

## 4. Terpenoids

Although volatiles only represent 10% of the propolis constituents, they account for the characteristic resinous odor and contribute to the pharmacological effects of propolis. As the major compounds among the volatile substances, terpenoids play an important role in distinguishing premium propolis from inferior or fake propolis and they exhibit antioxidant, antimicrobial, and other biological activities.

Monoterpenes isolated from propolis include acyclic, monocyclic, dicyclic monoterpenes and their derivatives. The primary acyclic and monocyclic monoterpenes are myrcenes, *p*-menthanes and cineoles, respectively. The dicyclic monoterpenes in propolis are classified into five groups: thujanes, caranes, pinanes, fenchanes and camphenes. Sesquiterpenes are the most abundant chemical components in propolis. According to the number of the rings, sesquiterpenes fall into four categories: acyclic, monocyclic, dicyclic and tricyclic. The main acyclic sesquiterpenes in propolis are the derivatives of farnesane. There are four types of monocyclic sesquiterpenes, five types of dicyclic sesquiterpenes and ten types of tricyclic sesquiterpenes in propolis. Cembrane, labdane, abietane, pimarane, and totarane are reported to be the major diterpenes in propolis, and some of these are proven to have a broad spectrum of pharmacological properties. The tetracyclic triterpenes in propolis are lanostanes and cycloartane and the pentacyclic triterpenes are oleanane, ursane and lupane.

One monoterpene (*trans*-β-terpineol) and three sesquiterpenes (γ-elemene, α-ylangene, valencene) with valuable biological activities were identified in Brazilian propolis [52]. In Turkish propolis, a few sesquiterpenes **119**–**123** were identified; and there was no direct evidence to determine the correct plant source of the each type of Turkish propolis [53]. Popova *et al.* identified the usual “Mediterranean” diterpenes in samples from Greece, together with some diterpenes (Table 2) that are deemed as characteristic oleoresin components of different Coniferae (mainly Pinaceae and Cupressaceae) plants [29], although their plant source was considered to be the Cupressaceae because Greek propolis contained ferruginol, totarol, oxygenated ferruginol and totarol derivatives, and sempervirol, which are typically found in Cupressaceae plant, but not in Pinaceae. Some triterpenes belonging to the lupane (**154**–**156**), lanostane (**157**–**158**), oleanane (**159**–**161**), ursane (**162**–**164**) and other types (**165**–**170**) were found in Brazilian, Cuban, Greek, Burmese and Egyptian propolis for the first time.

**Table 2:** Terpenes identified in propolis since 2000.

No.	Chemical Name	Geographical Location	Reference
Monoterpenes			
112	*trans*-β-Terpineol	Greece	[54]
113	Linalool	Brazil	[52]
114	Camphor	Iran	[55]
Sesquiterpenes			
115	Junipene	Greece	[54]
116	γ-Elemene	Brazil	[52]
117	α-Ylangene	Brazil	[52]
118	Valencene	Brazil	[52]
119	8-β*H*-Cedran-8-ol	Turkey	[53]
120	4-β*H*,5α-Eremophil-1(10)-ene	Turkey	[53]
121	α-Bisabolol	Turkey	[23]
122	α-Eudesmol	Turkey	[23]
123	α-Cadinol	Turkey	[23]
124	Patchoulene	Indonesia	[56]
Diterpenes			
125	Manoyl oxide	Greece	[57]
126	Ferruginol	Greece	[57]
127	Ferruginolone	Greece	[57]
128	2-Hydroxyferruginol	Greece	[57]
129	6/7-Hydroxyferruginol	Greece	[57]
130	Sempervirol	Greece	[57]
131	Abietic acid	Greece	[57]
132	18-Succinyloxyabietadiene	Greece	[57]
133	18-Succinyloxyhydroxyabietatriene	Greece	[57]
134	18-Hydroxyabieta-8,11,13-triene	Greece	[57]
135	Imbricataloic acid	Greece	[57]
136	Imbricatoloic acid	Greece	[57]
137	Diterpenic acid	Greece	[57]
138	Neoabietic acid	Greece	[57]
139	Labda-8(17),12,13-triene	Greece	[57]
140	Hydroxydehydroabietic acid	Greece	[57]
141	Dihydroxyabieta-8,11,13-triene	Greece	[57]
142	13(14)-Dehydrojunicedric acid	Greece	[57]
143	Dehydroabietic acid	Greece	[57]
144	18-Hydroxyabieta-8,11,13-triene	Greece	[57]
145	Junicedric acid	Greece	[29]
146	14,15-Dinor-13-oxo-8(17)-labden-19-oic acid	Greece	[29]
147	*tran*-Communal	Greece	[29]
148	Palmitoyl isocupressic acid	Greece	[29]
149	Oleoyl isocupressic acid	Greece	[29]
150	13-Hydroxy-8(17),14-labdadien-19-oic acid	Greece	[29]
151	15-Oxolabda-8(17),13(*E*)-dien-19-oic acid	Greece	[29]
152	Pimaric acid	Greece	[29]
153	Totarolone	Greece	[29]
Triterpenes			
154	Lupeol alkanoates	Brazil	[58]
155	Lupeol	Brazil	[58]
156	Lupeol acetate	Cuba	[59]
157	Lanosterol acetate	Egypt	[35]
158	Lanosterol	Cuba	[59]
159	Germanicol acetate	Cuba	[59]
160	Germanicol	Cuba	[59]
161	β-Amyrin acetate	Cuba	[59]
162	β-Amyrone	Cuba	[59]
163	α-Amyrin acetate	Cuba	[59]
164	α-Amyrone	Cuba	[59]
165	24-Methylene-9,19-ciclolanostan-3β-ol	Brazil	[58]
166	(22*Z*,24*E*)-3-Oxocycloart-22,24-dien-26-oic acid	Burma	[60]
167	(24*E*)-3-Oxo-27,28-dihydroxycycloart-24-en-26-oic acid	Burma	[60]
168	3,4-seco-Cycloart-12-hydroxy-4(28),24-dien-3-oicacid	Greece	[29]
169	Cycloart-3,7-dihydroxy-24-en-28-oic acid	Greece	[29]
170	3-Oxo-triterpenic acid methyl ester	Egypt	[61]

## 5. Phenolics

Brazilian green propolis is rich in phenylpropanoids including cinnamic acid, p-coumaric acid, caffeic acid, ferulic acid and their derivatives. Among these substances, prenylated cinnamic acids turn out to be a salient chemical feature and have a consanguineous bearing on antimicrobial activity of green propolis. In recent years, researchers identified a series of phenylpropanoid derivatives **171**–**180** in Brazilian propolis. Meanwhile, some caffeic acid derivatives **182**–**183** and isoferulic acid derivative **184** were also identified in poplar propolis by GC-MS. Chlorogenic acid is abundant in Brazilian propolis of floral origin from *Citrus* spp. [62]. Three quinic acid derivatives **185**–**187** were identified in this type of propolis. 

Another class of phenolics, stilbenes, are not very common in plants. In 2010, Petrova *et al.* identified two geranylstilbenes; schweinfurthin A (188) and schweinfurthin B (**189**) in propolis produced in Kenya. *Macaranga schweinfurthii* is the only plant source of these two geranylstilbenes to this date [37]. In 2012, another stilbene, 5-farnesyl-3'-hydroxyresveratrol (**190**) was identified in Solomon Island propolis, which is also present in Macaranga plants [31]. These results suggest that *Macaranga* is probably the plant source of the propolis from Kenya and Solomon Island. However, many stilbenes **191**–**202**, especially prenylated stilbenes, were identified in Australian Kangaroo Island propolis, which makes this type of propolis a stronger scavenging activity towards DPPH free radical than Brazilian propolis [63], suggesting the source of stilbenes is not limited to only a few plants.

Lignans as main chemical compounds in tropical propolis have attracted a worldwide research interest. In the past 12 years, researchers identified three lignans **206**–**208** in Kenyan and Brazilian propolis. As shown in the Table 3, other phenolic compounds and derivatives were identified in propolis from Brazil (**209**–**219**), Indonesia (**220**–**229**), France (**230**), Iran (**231**–**239**) and Malta **(240**–**241)**. Among these chemicals, nemorosone (**215**) is the exclusive and principal component of *Clusia rosea* floral resins, indicating that *Clusia* spp. is the plant origin of the brown propolis [64]. Tschimgin (**232**), tschimganin (**233**), ferutinin (**236**), tefernin (**237**) identified in Iranian propolis are the characteristic compositions of the *Ferula* species, which is considered as another plant source of Iranian propolis besides poplar. 

**Table 3:** Phenolics identified in propolis since 2000.

No.	Chemical Name	Geographical Location	Reference
Phenylpropanoids			
171	*cis*-3-Methoxy-4-hydroxycinnamic acid	Brazil	[65]
172	*trans*-3-Methoxy-4-hydroxycinnamic acid	Brazil	[65]
173	3-Prenyl cinnamic acid allyl ester	Brazil	[66]
174	*p*-Methoxycinnamic acid	Brazil	[66]
175	Dihydrocinnamic acid	Brazil	[66]
176	3-Prenyl-4-hydroxycinnamic acid	Brazil	[67]
177	3,5-Diprenyl-4-hydroxycinnamic acid	Brazil	[67]
178	3-Methyl-2-butenyl isoferulate	Brazil	[66]
179	3-Methyl-3-butenyl caffeate	Brazil	[66]
180	Hexadecyl caffeate	Brazil	[66]
181	Methyl(*E*)-4-(4'-hydroxy-3'-methylbut-(*E*)-2'-enyloxy) cinnamate	Australia	[63]
182	Tetradecenyl caffeate (isomer)	Egypt	[35]
183	Tetradecenyl caffeate	Egypt	[35]
184	2-Methyl-2-butenyl ferulate	Uruguay	[68]
Chlorogenic acids			
185	4-Feruoyl quinic acid	Brazil	[62]
186	5-Ferruoyl quinic acid	Brazil	[33]
187	3,4,5-tri-*O*-Caffeoylquinic acid	Brazil	[69]
Stilbenes			
188	Schweinfurthin A	Kenya	[37]
189	Schweinfurthin B	Kenya	[37]
190	5'-Farnesyl-3'-hydroxyresveratrol	Solomon Island	[31]
191	5,4'-Dihydroxy-3'-methoxy-3-prenyloxy-*E*-stilbene.	Australia	[63]
192	3,5,3',4'-Tetrahydroxy-2-prenyl-*E*-stilbene	Australia	[63]
193	3,5,4'-Trihydroxy-3'-methoxy-2-prenyl-*E*-stilbene	Australia	[63]
194	5,3',4'-Trihydroxy-3-methoxy-2-prenyl-*E*-stilbene	Australia	[63]
195	5,4'-Dihydroxy-3,3'-dimethoxy-2-prenyl-*E*-stilbene	Australia	[63]
196	5,4'-Dihydroxy-3-prenyloxy-*E*-stilbene	Australia	[63]
197	3',4'-Dihydroxy-*E*-stilbene	Australia	[63]
198	3',4'-Dihydroxy-3,5-dimethoxy-*E*-stilbene	Australia	[63]
199	Diprenylated dihydrostilbene	Australia	[63]
200	3,5-Dihydroxy-2-prenyl-*E*-stilbene	Australia	[63]
201	4-Prenyldihydroresveratrol	Australia	[63]
202	3-Prenylresveratrol	Australia	[63]
203	(+)-Pinoresinol dimethyl ether	Brazil	[44]
204	(+)-Pinoresinol	Brazil	[44]
205	(+)-Syringaresinol	Brazil	[44]
Lignans			
206	Tetrahydrojusticidin B	Kenya	[37]
207	6-Methoxydiphyllin	Kenya	[37]
208	Phyllam ricin C	Kenya	[37]
Other phenolics			
209	8-(Methyl-butanechromane)-6-propenoic acid	Brazil	[70]
210	3-Hydroxy-2,2-dimethyl-8-prenylchromane-6-propenoic acid	Brazil	[70]
211	2,2-Dimethyl-8-prenylchromene-6-propenoic acid	Brazil	[70]
212	2,2-Dimethylchromene-6-propenoic acid	Brazil	[70]
213	2,2-Dimethyl-6-carboxyethnyl-2*H*-1-benzopyran	Brazil	[70]
214	2,2-Dimethyl-6-carboxyethenyl-8-prenyl-2*H*-1-benzopyran	Brazil	[70]
215	Nemorosone	Brazil	[9]
216	7-epi-clusianone	Brazil	[9]
217	Xanthochymol	Brazil	[9]
218	Gambogenone	Brazil	[9]
219	Hyperibone A	Brazil	[71]
220	5-Pentadecylresorcinol	Indonesia	[72]
221	5-(8'*Z*,11'*Z*-Heptadecadienyl)-resorcinol	Indonesia	[72]
222	5-(11'*Z*-Heptadecenyl)-resorcinol	Indonesia	[72]
223	5-Heptadecylresorcinol	Indonesia	[72]
224	1,3-Bis(trimethylsilylloxy)-5,5-proylbenzene	Indonesia	[56]
225	3,4-Dimethylthioquinoline	Indonesia	[56]
226	4-Oxo-2-thioxo-3-thiazolidinepropionic acid	Indonesia	[56]
227	D-glucofuranuronic acid	Indonesia	[56]
228	Dofuranuronic acid	Indonesia	[56]
229	3-Quinolinecarboxamine	Indonesia	[56]
230	Baccharin	France	[73]
231	Suberosin	Iran	[55]
232	Tschimgin	Iran	[55]
233	Tschimganin	Iran	[55]
234	Bornyl *p*-hydroxybenzoate	Iran	[55]
235	Bornyl vanillate	Iran	[55]
236	Ferutinin	Iran	[55]
237	Tefernin	Iran	[55]
238	Ferutinol *p*-hydroxybenzoate	Iran	[55]
239	Ferutinol vanillate	Iran	[55]
240	2-Acetoxy-6-*p*-methoxybenzoyl jaeschkeanadiol	Malta	[74]
241	2-Acetoxy-6-*p*-hydroxybenzoyl jaeschkeanadiol	Malta	[74]

## 6. Sugars

The question about the origin of sugars in propolis has not been solved yet. Nectar and honey are thought to be the sources of glucose, fructose and sucrose. Others suggest that they come from hydrolyzed flavonoid glycosides in propolis. In addition, mucilages containing numerous sugars, sugar alcohols and acids were listed among potential propolis sugar sources by Crane [75]. In the propolis originated from the Canary Islands and Malta, many sugars, sugar alcohols and uronic acids were identified, supporting the claim that plant mucilages were the source of these compounds [74]. In Egyptian propolis, many sugars, sugar alcohols and uronic acids were identified by GC-MS. Among these substances, galactitol, gluconic acid, galacturonic acid and 2-*O*-glycerylgalactose were identified in propolis for the first time [61]. 

## 7. Hydrocarbons

Hydrocarbons are other basic components of propolis. In recent years, alkanes, alkenes, alkadienes, monoesters, diesters, aromatic esters, fatty acids and steroids have been identified in many types of propolis such as Egyptian propolis [35], Brazilian propolis [65] and Anatolian propolis [76]. Comparing the compositions of Brazilian propolis waxes and comb waxes which were produced by the same colony, no difference was found to allow a distinction, suggesting a common origin for both wax sources [77]. This result not only illustrates that propolis waxes are secreted by bees [78], but also indicates that the composition of propolis waxes and comb waxes is only dependent on genetic factors of the bees, not plant sources.

## 8. Mineral Elements

Trace elements (Ca, K, Mg, Na, Al, B, Ba, Cr, Fe, Mn, Ni, Sr and Zn) and toxic elements (As, Cd, Hg and Pb) were discovered by atomic emission/absorption spectrometry in propolis samples collected from different Croatian regions [79]. Br, Co, Cr, Fe, Rb, Sb, Sm and Zn were identified in different Argentinean propolis by neutron activation analysis. These studies show that the trace element profiles can be useful for propolis identification according to their location [80].

## 9. The Chemical Categories Reported in Propolis

**Figure 3:**
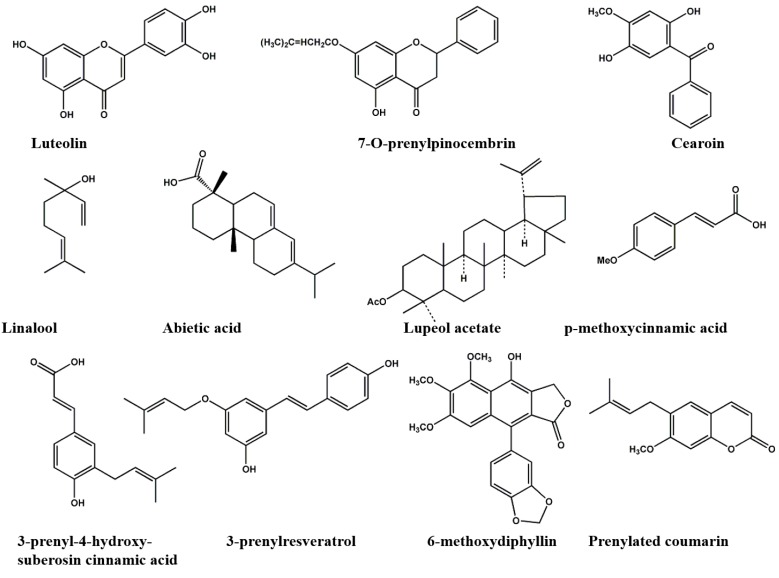
Representative chemical components identified in propolis since 2000.

The chemical categories reported in propolis during 2000 and 2012 are summarized in Figure 3 and Table 4, indicating consistency with the categories reported previously (Figure 1). It is well recognized that the chemical composition of herbal medicines are affected by many environmental factors while maintaining their genetic characteristics [81]. Similar effects to propolis can be expected from environmental factors. However, bee species needs to be considered together with geographical factors and plant sources.

**Table 4:** The chemical categories reported in propolis since 2000.

Chemical Category	Example Compound	Geographical Origin	Plant Source	Bee Species	References
Flavonoids	Luteolin	Australia, Brazil, Burma, Canada, Chinese, Cuba, Egypt, Greece, Japan, Kenya, Mexico, Nepal, Poland, Portugal, Solomon Island, Taiwan	*Populus, Macaranga, Dalbergia*	*Apis mellifera*	[26,31,34,36,37,38,39,41,42,43,44,45,46,47,61]
Prenylated flavanones	7-*O*-prenylpino-cembrin	Greece, Japan		*Apis mellifera*	[39,42]
Neo-flavonoids	Cearoin	Nepal	*Dalbergia*	*Apis mellifera*	[50]
Monoterpenes Sesquiterpenes Diterpenes	Linalool abietic acid	Brazil, Greece, Indonesia, Iran, Malta, Turkey	*Ferula Pinaceae Cupressaceae*	*Apis mellifera*	[37,52,53,55,56,74]
Triterpenes	Lupeol acetate	Burma, Brazil, Cuba, Egypt, Greece		*Apis mellifera*	[29,35,58,59,60]
Phenylpropanoids and esters	*p*-Methoxycinnamic acid	Australia, Brazil, Egypt, Uruguay	*Citrus*	*Apis mellifera*	[61,63,66,68]
Prenylated phenylpropanoids	3-Prenyl-4-hydroxycinnamic acid	Brazilian Green propolis	*Baccharies*	Africanized *Apis mellifera*	[67]
Stilbenes and prenylated stilbenes	3-Prenylresveratrol	Australia, Brazil, Greece, Indonesia, Kenya	*Macaranga*	*Apis mellifera*	[31,37,44,63,72]
Lignans	6-Methoxydiphyllin	Kenya		*Apis mellifera*	[37]
Coumarins	Prenylated coumarin suberosin	Iran		*Apis mellifera*	[55]

## 10. Bee Species and Propolis

We propose that species, subspecies and varieties of bees have a major impact on the chemical components and quality of propolis. The genus *Apis* contains 10 generally recognized species. Honeybee, *A. mellifera*, is widely spread in Europe, Ural Mountains, Africa, and Asia. All other recognised *Apis* species are of Asian distribution. About 25 subspecies have been recognized for *A. mellifera*, based on morphometry, behaviour and biogeography [82], belonging to three or four major subspecies groups [83]. 

The most popular species of honeybee is the European honeybee, *Apies mellifera*. It has been shown that varieties of bee affect the antibacterial activity of propolis collected from the same apiary; *A. mellifera carnica* hives showed weaker antibacterial activity than that of *A. mellifera anatolica* and *A. mellifera caucasica.* The three honeybee races used neither the same nor the single plant source [23]. In another type of propolis, geopropolis, produced by stingless bee species, *Melipona scutellaris*, benzophenones, but no flavonoids, have been identified as the major compounds [84]; However, geopropolis produced by *Melipona fasciculate* contains high concentrations of polyphenols, flavonoids, triterpenoids, saponins, and even tannins [85]. 

Although different species of honeybee prefer different plants, the chemical profile of propolis that is produced by the same species is not always same. Brazilian green and red propolis both originate from Africanized *A. mellifera* [65,86], but these propolis are rich in prenylated phenylpropanoids and isoflavonoids respectively. The differences are due to the plants, namely *B. dracunculifolia* and *Dalbergia ecastophyllum,* which are used by bees as resin sources*.* In cerumen propolis from stingless bees (*Tetragonula carbonaria*), *C*-methylated flavanones, terpenic acids and phenolic acids, such as gallic acid, diterpenic acids of pimaric and abietic type are the predominant chemicals, but it lacks the characteristic flavonoids and prenylated phenolics found in propolis from honeybees species in Australia [87,88]. Therefore, the variant chemical composition of propolis depends on the bees’ preferences of botanical sources and the species and varieties of bees [89,90,91]. 

## 11. The Geographical Origins of Propolis

Propolis collected from many countries have demonstrated chemical profiles similar to the poplar type propolis: China [92], Korea, Croatia [93], different regions of Taiwan [43,94,95], New Zealand [96] and Africa [35]. Poplar tree (*Populus nigra* L. and *P. alba* L) is common in Europe, and is used to name the common type of propolis that is rich in flavonoids and phenylpropanoids. However, flavonoids are not restricted to poplar; furthermore, in areas where poplars are not native plants, such as Australia and equatorial regions of South America, bees will seek other plants to produce propolis, which contain the flavonoids of the poplar type propolis [36].

Propolis from the tropical zone, Brazilian green and red propolis, are respectively rich in prenylated derivatives of *p*-coumaric acid, and some isoflavonoids that are different from the ones found in poplar type propolis [3,97]. In addition, propolis from Solomon Island, Burma, Greek, Japan are characterized by the geranylated and prenylated flavonoids (Table 1).

## 12. The Plant Sources of Propolis

The current opinion is that propolis is collected from resins of trees such as poplars and conifers, and therefore propolis is sometimes classified after the name of the source plant [2,3,4]. The plant source is identified by observing the collection activities of bees, and comparing the chemical profiles of propolis and plant materials. Other researchers found that honeybees collect plant material by cutting fragments of vegetative tissues, so the anatomical characteristics of plant tissue in the propolis can be used as evidence of propolis origin [65].

As mentioned in the last section, *Populus* species are considered to be the main plant origin of propolis all over the world, especially in the temperate zone. Most propolis collected from Europe, North America, non-tropical region of Asia, New Zealand [3] and even Africa (mainly the east area of Nile Delta region) [35] contains the characteristic poplar chemical profile: high level of flavanones, flavones, low phenolic and their esters [98]. 

In the tropical and subtropical area, there are few poplar trees. Honeybees have to search for new plant source for propolis. For the propolis collected from southeast of Brazil, *Baccharis dracunculifolia* turns out to be the main botanical source [66,99]. Artepillin C as the salient chemical composition makes it easy to distinguish this propolis from other types of propolis. It is reported that propolis from Venezuela, Amazon and Cuba contains prenylated benzophenones, which is originated from the exudates of *Clusia* flower [9,100]. 

*Macaranga* plants have been demonstrated to be the plant source of Taiwan [95], Okinawan [101] that was classified as Pacific propolis [3]. High concentration of diterpenoids in Mediterranean propolis may originate from *Cupressus* plants for Sicilian, Cretan propolis [29] and Maltese propolis [74], *Pinus* plants for Greek propolis [39]. In Kangaroo Island (Australia), bees collect propolis from the sticky exudate on the stem shoots and seed pods of an endemic Australian plant, *Acacia paradoxa* [45]. Red Brazilian propolis and Nepalese propolis have various biologically active neoflavonoids that primarily come from the genus *Dalbergia* [24,50].

However, some of plant sources are just surmised by observing the bees’ foraging behaviors, not comparing chemical identity of secondary plant metabolites in propolis and in the plant source. For example, *Eucalyptus* species are considered as the source plant in Australia, south Anatolia (Turkey) [102], Ismailia (Egypt) [61] and Brazil, but no real proof has been presented for this origin. Therefore, it still needs further study to compare chemical compounds in propolis and the plants, in order to confirm the exact botanic origin.

## 13. Summary and Future Perspectives

The biological activities of propolis are attributed to a variety of major chemical constituents including phenolic acids, phenolic acid esters, flavonoids, and terpenoids, such as CAPE, artepillin C, caffeic acid, chrysin, and galangin quercetin, apigenin, kaempferol, pinobanksin 5-methyl ether, pinobanksin, pinocembrin, pinobanksin 3-acetate. 

Over 500 compounds have been identified in propolis from many countries up to 2012. They belong to flavonoids, phenylpropanoids, terpenoids, stilbenes, lignans, coumarins and their prenylated derivatives. However, other common chemical components such as alkaloids, iridoids have not been reported in propolis. This characteristic is often explained by the plant sources.

We recommend that bee varieties and subspecies need to be considered together with geographical factors and plant species around the beehive in future studies on propolis. The priorities of future research lie on the influence of species and behaviour on propolis, together with feeding experiments to identify the plant part source, which will advance our understanding of the chemistry and quality of propolis, as well as honey bee biology. Characterization of propolis from various locations and plant sources is warranted to define acceptable quantitative standards for different types of propolis. Furthermore, the biological activities of each type of propolis need to be correlated with their chemical composition, and eventually, standardized products should be used in clinical studies. 
